# Self-Inflicated Cardiac Injury by Multiple Sewing Needles

**DOI:** 10.4274/balkanmedj.2017.0585

**Published:** 2018-03-15

**Authors:** Özgür Altınbaş, Sümeyye Fatma Özer, Abdurrahman Şeramet, Ömer Tanyeli, Niyazi Görmüş

**Affiliations:** 1 Department of Cardiovascular, Necmettin Erbakan University Meram School of Medicine, Konya, Turkey; 2 Department of Cardiology, Necmettin Erbakan University Meram School of Medicine, Konya, Turkey

Although penetrating injuries by needles is a rare clinical situation, they can lead to mortal and morbid conditions due to tamponade, infection, embolism, valve disorders and thrombus (1). There might be a clinically silent period for many years but pericarditis, tamponade or endocarditis can develop over time. Therefore, strict follow-up is important in those patients (2).

A 25-year-old male prisoner was admitted to our clinic with a complaint of nonspecific chest pain. After a detailed history, he confided that he intentionally inserted sewing needles into himself because he wanted to have surgery in his hometown hospital.

His vital signs and laboratory findings were stable. Chest radiography revealed nearly 20 needles within the cardiac silhouette and the thorax (Figure 1).

Contrasted thorax computed tomography also revealed multiple foreign metallic objects reached to pericardium, myocardium and thorax and fibrosis due to needles (Figure 2).

He had a normal systolic function on trans-thoracic echocardiographic examination and, because of self-restriction of small bleeding foci, there was no pericardial effusion that might lead to heart compression.

After getting informed consent we decided to undertake direct surgery at our center, but the patient didn't accept'. The patient suffered from chest pain during the 3-month follow-up period.

Intracardiac lesions caused by the insertion of needles in an attempt to inflict self-injury have been described in only a few cases. Detailed anamnesis is recommended in such patients (3). Although there might be an asymptomatic period, if left untreated, the sharp nature of needles enables them to rapidly migrate through the tissues, leading to serious complications, including cardiac tamponade, hemothorax and pneumothorax; thus, delayed diagnosis is associated with poor outcome (1). In most cases, early surgery is recommended to prevent migration and further anatomic damage. However, some reports have shown that old wounds and asymptomatic foreign bodies can be treated conservatively because, with time, most foreign bodies become safely encysted and benign (4).

In our case, although we recommended surgery, the patient did not accept. Because of the risk of late complications, a strict follow-up is necessary for such patients.

## Figures and Tables

**Figure 1:**
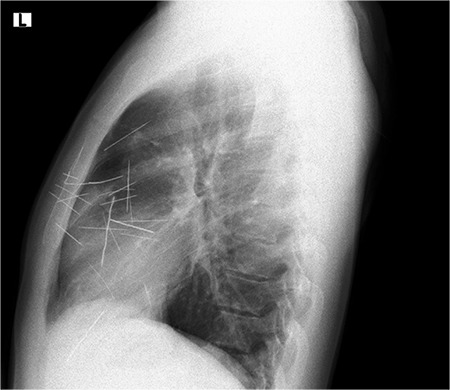
Thin, linear, metallic opacities superpose to sternum, middle and anterior mediastinum and diaframe in chest lateral X-ray.

**Figure 2:**
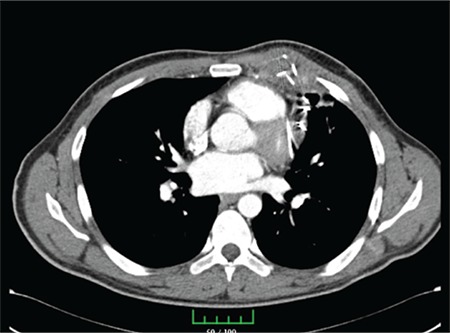
Metallic linear foreign material densities in chest wall, soft tissues and pericardial level in left parasternal area in thorax computed tomography image-mediastinum window.
